# Supplementary material for: Endometrial Inflammation and Impaired Spontaneous Decidualization: Insights into the Pathogenesis of Adenomyosis
Source: Int J Environ Res Public Health. 2023 Feb 20;20(4):3762. doi: 10.3390/ijerph20043762 (PMC9964052; doi:10.3390/ijerph20043762)
Supplement: Supplementary file 1 [file ijerph-20-03762-s001.zip › Supplementary Table S1.pdf]

Supplementary Table S1. The key molecules associated with fibrosis and their functions in adenomyosis.

Official Symbol	Official Full Name	Summary	Refs.
TGFB	transforming growth factor-beta	TGF-β acts as a complex and multifaceted regulator of various reproductive processes including folliculogenesis, oocyte maturation, ovulation, maternal-embryo interactions, implantation, decidualization, and embryonic development. Platelet-derived TGF-β and its downstream Smad signaling pathway play a critical role in the development of adenomyosis through various cellular processes inducing EMT, FMT, SMM, and fibrosis in a mouse model of adenomyosis. In contrast, there are inconsistent data on the expression of TGF-β and its receptors, indicating that they are not involved in the development of adenomyosis. Distinct TGF-β expression patterns may be implicated with the spatial and temporal expression patterns in the subtypes of adenomyosis. In addition, TGFβ signaling has been reported to play an important role in the decidualization process. Evidence from human studies showed that TGFβ1 is secreted from human endometrial stromal cells during in vitro decidualization and recombinant TGFβ1 promotes the decidualization process. In contrast, other studies demonstrated that TGFβ1 inhibits the expression of the decidualization-related genes, including PRL, IGFBP-1, and TF in human endometrial stromal cells, suggesting that female reproduction may be adversely affected by overexpression of TGF-β1. Also, inhibition of TGF-β1 improved pregnancy	[11,15,33,36-41]

		outcomes (i.e., live birth numbers and rates and survival and pup mortality rate) by restoring endometrial receptivity through increasing LIF expression in mice with adenomyosis. Contradictory results have been observed regarding the effects of TGFβ1 on decidualization, which may be associated with differences in animal species used or experimental conditions utilized in different studies.	
OXTR	oxytocin receptor	Oxytocin receptor belongs to the G-protein coupled receptor family and acts as a receptor for oxytocin. The expression level of oxytocin receptor in the fundus of adenomyosis uteri was higher than that in the control uteri, which can lead to dysmenorrhea as a result of increased uterine contractions. In addition, inhibition of oxytocin improved endometrial receptivity by decreasing endometrial COX-2 activity and PGF2α production. OXTR overexpressed in adenomyosis causes impaired decidualization, resulting in implantation failure.	46
NTRK2	neurotrophic receptor tyrosine kinase 2, also known as TrkB	Nerve growth factor (NGF) and brain-derived neurotrophic factor (BDNF) induce neuropathic pain through TrkA and TrkB receptors, respectively. TrkB activates the MAPK pathway upon neurotrophin binding. Elevated TrkB expression in the endometrium of adenomyosis correlates with the severity of dysmenorrhea. BDNF upregulates MMP-9 expression through activating the phosphatidylinositol 3-kinase pathway and promotes blastocyst outgrowth via TrkB.	47,48

NOTCH1	notch receptor 1	The expression of Notch1 is upregulated in the ectopic endometrium of adenomyosis compared with normal endometrium. Notch1 is involved in the EMT pathway in adenomyosis and plays a crucial role in decidualization and implantation.	49
