# Supplementary material for: Endometrial Inflammation and Impaired Spontaneous Decidualization: Insights into the Pathogenesis of Adenomyosis
Source: Int J Environ Res Public Health. 2023 Feb 20;20(4):3762. doi: 10.3390/ijerph20043762 (PMC9964052; doi:10.3390/ijerph20043762)
Supplement: Supplementary file 1 [file ijerph-20-03762-s001.zip › Supplementary Table S2.pdf]

Supplementary Table S2. The key molecules associated with angiogenesis and their functions in adenomyosis.

Official Symbol	Official Full Name	Summary	Refs.
HIF1A	hypoxia inducible factor 1 subunit alpha	HIF-1 functions as a master regulator of homeostatic response to hypoxia by regulating energy metabolism, metabolic adaptation to hypoxia, angiogenesis, proliferation, and apoptosis. HIF-1 plays a key role in embryonic vascularization, tumor angiogenesis and pathophysiology of ischemic disease. Expression of HIF-1A in the ectopic endometrium of adenomyosis was positively correlated with microvessel density and VEGF expression. HIF-2α exerts an important function on angiogenesis and decidualization via downregulating the expression of HOXA10 and HOXA11. HIF may be widely involved in the pathogenesis of adenomyosis.	[51,53,54]

VEGFA	vascular endothelial growth factor A	VEGF induces proliferation and migration of vascular endothelial cells, and is essential for both physiological and pathological angiogenesis. VEGF stimulates blood and lymph vascular angiogenesis in the ectopic endometrium of patients with adenomyosis. The expression level of VEGF protein in the ectopic endometrium of patients with adenomyosis was higher than that in the eutopic endometrium, and the expression level of VEGF in the eutopic endometrium was higher than that in the normal endometrium of healthy controls. The ectopic and eutopic endometrium of adenomyosis patients contain a higher number of alpha-smooth muscle actin (α-SMA)-negative vascular smooth muscle cells than those in the normal endometrium. Therefore, blood vessels in adenomyosis are irregular, immature, defective, and fragile, so they may be different from physiological cyclic angiogenesis in normal endometrium. Moreover, VEGF stimulates CXCL1 expression in human endometrial epithelial cells and induces vascular endothelial cell migration via the NF-κB signaling pathway. In addition, estradiol induces pro-angiogenic activity in vascular endothelial cells by activating VEGF signaling in endometrial epithelial cells [56]. VEGF induces macrophage recruitment and M2 polarization, suggesting that VEGF functions as a linking hub between the decidualization	[53-60]

		network and angiogenesis. VEGF also promotes a suitable microenvironment for embryonic implantation and development by improving endometrial receptivity. Like HIF, VEGF is a key molecule involved in the pathogenesis of adenomyosis, particularly in the angiogenesis and decidualization processes. Overexpression of HIF and VEGF contributes to the creation of immature vessels characterized by frgile and permeable vessels, resulting in decidualization failure.	
CXCL1	C-X-C motif chemokine ligand 1	CXCL1 is a secreted growth factor that signals through the G-protein coupled receptor, CXC receptor 2. This protein plays a role in inflammation and as a chemoattractant for neutrophils, monocytes, and macrophages. The expression of CXCL1 was increased in decidualized endometrial stromal cells of patients with adenomyosis. CXCL1 is involved in the processes of decidual angiogenesis via the VEGF-A pathway. Furthermore, CXCL1 is associated with diverse organs tissue remodeling and fibrosis through enhancing leukocyte recruitment and promoting inflammation.	[4,61,62]

ANXA2	annexin A2	Annexin 2 (ANXA2), a calcium-dependent phospholipid-binding protein, contributes to various functions such as cellular growth, signal transduction, anti-inflammation, and angiogenesis, which promotes tissue repair. ANXA2 as an estrogen-responsive protein is upregulated in the ectopic endometrium of adenomyosis compared with its eutopic counterpart or the normal endometrium. ANXA2 promotes endometrial cell invasion and EMT through activating HIF-1α/VEGF-A and β-catenin/T-cell factor (TCF) pathway. Moreover, ANXA2 stimulates endometrial tissue growth, invasion, angiogenesis, and hyperalgesia in an adenomyosis nude mice model. Additionally, ANXA2 expression in the ectopic endometrium of women with adenomyosis was positively correlated with the severity of dysmenorrhea. ANXA2 also contributes to the decidualization process of human endometrial cells. Decreased expression of ANXA2 leads to decidualization failure, resulting in various obstetric disorders, such as preeclampsia. The causal relationship between ANXA2 overexpression and decidualization failure in adenomyosis is still unknown.	[63-67]

F3	coagulation factor III, also known as tissue factor	TF, a cofactor for factor VII/VIIa, is the primary initiator of blood coagulation. TF is produced from activated platelets and monocytes under procoagulant and proinflammatory stimuli. Furthermore, TF is expressed in endometrial stromal cells by progesterone and is involved in angiogenesis and decidualization. In non-fertile cycle, progesterone withdrawal causes menstrual-associated bleeding via decreased TF production. Local thrombin generation induced by endometrial hemorrhage downregulates the expression of progesterone receptors in decidual cells, resulting in upregulation of inflammatory cytokines associated with inflammation and hypoxia. Excessive angiogenesis induced by hypoxia leads to large fragile vessels. TF expression in the adenomyotic endometrium is associated with heavy menstrual bleeding and dysmenorrhea. In addition, the expression levels of other pro-angiogenic markers (e.g., vWF and NF-κB nuclear p65) have also been reported to be associated with the severity of bleeding associated with adenomyosis.	[50,68-70]
MMP-2, MMP-9	matrix metallopeptidase 2, matrix metallopeptidase 9	Members of the matrix metalloproteinase (MMP) gene family are involved in multiple pathways including roles in the endometrial menstrual breakdown, regulation of vascularization, and cancer metastasis. The expression levels of MMP-2 and MMP-9 were higher in the ectopic and	[18,50,56,71-73]

		eutopic endometrium of adenomyosis than in normal endometrium. There was a positive correlation between MVD and MMP-2 or MMP-9 expression. Increased expression of MMPs in endometrial tissue may play an important role in the development of adenomyosis through the processes of invasion and angiogenesis. Increased expression of MMP induced by sex hormone withdrawal promotes cyclic shedding of the endometrium. Decidual cells and uterine NK cells primarily produce MMP-9 and MMP-2, respectively. The immune cell recruitment provides degradative enzymes (e.g., MMPs), which lead to menstrual bleeding.	
IL10	interleukin 10	IL-10 is a cytokine produced primarily by monocytes and has immunoregulatory effects, including anti-inflammatory, anti-angiogenic, and immunostimulatory properties. IL-10 down-regulates the expression of major histocompatibility complex class II antigens on macrophages and limits Th1 inflammatory response. IL-10 also blocks the NF-κB signaling. The expression level of IL-10 was decreased in the ectopic and eutopic endometrium of women with adenomyosis compared with control endometrium. IL-10 increased HOXA10 expression by inducing the phosphorylation of STAT3 in endometrial cell line. IL-10 deficiency impairs decidualization and endometrial receptivity in women with	[50,74]

		adenomyosis via downregulating HOXA10 expression. IL-10 may play critical roles in decidualization and embryo implantation and contribute to the pathogenesis and pathophysiology of adenomyosis.	
CDH 1	cadherin 1 also known as E-cadherin	E-Cadherin is implicated in cell-cell adhesion and has a potent anti-angiogenic activity. The expression level of E-cadherin was decreased in the ectopic and eutopic endometrium of women with adenomyosis compared with control endometrium. E-cadherin was decreased in ectopic endometrium and eutopic endometrium compared with control endometrium. Downexpression of E-cadherin in the endometrium may lead to impaired endometrial receptivity possibly through upregulation of MMP-2 and -9 expression.	[50,75]
SLIT 2	slit guidance ligand 2	SLIT2 is a ligand for the Robo family of immunoglobulin receptors. The SLIT-ROBO system maintains vascular homeostasis through tight regulation of endothelial cell migration, proliferation, and angiogenesis. The expression level of SLIT in the ectopic endometrium of women with adenomyosis was higher than that in normal endometrium, and was associated with the severity of dysmenorrhea. In addition, aberrant SLIT-ROBO signaling plays a role in impaired endometrial receptivity by causing decidualization failure. In addition, the expression level of SLIT	[52,76-79]

		has been reported to be increased in endometriosis.	
KISS1	KiSS-1 metastasis suppressor	KISS1 is a metastasis suppressor gene that inhibits chemotaxis, motility, and invasion through regulation of MMPs. The expression level of KISS1 protein was higher in the ectopic as compared with the eutopic endometrium, and they were higher than that from women without adenomyosis. KISS1 inhibited cell motility in human decidual stromal cells through downregulating phosphorylation of focal adhesion kinase (FAK) and steroid receptor coactivator (Src) tyrosine kinases. KISS1 has a potential inhibitory role on decidualization and embryo implantation.	[79,80]
CEBPB	CCAAT enhancer binding protein beta, also known as C/EBPβ	C/EBPβ is a transcription factor that regulates cell proliferation, differentiation, and metabolism. C/EBPβ regulates human endometrial stromal cell growth and differentiation through cyclins E-cdk2 and STAT3 pathways. The expression level of C/EBPβ was increased in the endometrium from women with adenomyosis compared with controls. C/EBPβ induces H3K27ac throughout the genome and upregulates the expression of many genes associated with decidualization, such as IGFBP-1 and PRL, via DNA hypomethylation.	[81-83]

STIP 1	stress induced phosphoprotein 1	STIP1 is upregulated in adenomyosis lesions and enhances the MMP-9 expression.	[84]
NDU FA13	NADH:ubiquinone oxidoreductase subunit A13 (NDUFA13), also known as retinoid-interferon (IFN)-induced mortality 19 (GRIM-19)	GRIM-19 controls cellular proliferation by inhibiting G1/S transition through blocking STAT3-driven transcription of genes involved in cell proliferation and apoptosis resistance. GRIM-19 has been reported to play a key role in angiogenesis, endometrial receptivity, decidualization, and embryo implantation by regulating adhesion, apoptosis, cytokine expression, and immune tolerance. The expression level of GRIM-19 is downregulated in the ectopic endometrium of adenomyosis compared with its eutopic counterpart or the normal endometrium. M2 macrophages suppress the expression of GRIM-19 in adenomyotic cells through inhibiting the TLR4 signalling. GRIM19 downregulation promotes adenomyosis progression by upregulating IL-1β expression through activating macrophage inflammasomes and by inducing VEGF expression through stimulating STAT3 signaling in endometrial cells.	[85-89]
CAV 1	caveolin 1	Caveolin-1 (CAV1) is the fundamental component of caveolae and is associated with endocytosis, migration, metabolism, autophagy, and signaling. CAV1 functions as a negative regulator of the Ras-p42/44 mitogen-activated kinase (MAPK) cascade and was previously recognized as a candidate	[90,91]

		tumor-suppressor gene. The expression level of CAV1 in the ectopic endometrium of adenomyosis patients was lower than that of paired eutopic endometrium or normal controls. Loss of CAV1 expression enhances cell growth, migration, and invasion of endometrial epithelial and stromal cells, suggesting that CAV1 plays a crucial role in the pathogenesis of adenomyosis. In addition, CAV1 is involved in the decidualization process through downregulating IGFBP1 expression.	
CSF2	colony stimulating factor 2, also known as GMCSF	GM-CSF drives macrophage recruitment and is involved in angiogenesis and tissue remodeling. GM-CSF is produced by endometrial epithelial cells especially during the secretory phase of the menstrual cycle. The expression level of GM-CSF is upregulated in the ectopic endometrium of adenomyosis compared with its eutopic counterpart.	[92,93]
