# Supplementary material for: Endometrial Inflammation and Impaired Spontaneous Decidualization: Insights into the Pathogenesis of Adenomyosis
Source: Int J Environ Res Public Health. 2023 Feb 20;20(4):3762. doi: 10.3390/ijerph20043762 (PMC9964052; doi:10.3390/ijerph20043762)
Supplement: Supplementary file 1 [file ijerph-20-03762-s001.zip › Supplementary Table S3.pdf]

Supplementary Table S3. The key molecules associated with inflammation and their functions in adenomyosis.

Official Symbol	Official Full Name	Summary	Refs.
IL6, IL8	interleukin 6, interleukin 8	IL-6 was identified as a candidate hub gene that was closely related to inflammatory pathway, chemotactic network, and immune response in adenomyosis. Activation of the IL-6-ERK/MAPK signalling has been associated with the proliferation of adenomyosis smooth muscle cells. Overexpressed IL-6 in adenomyosis may suppress the decidualization process by negatively regulating HoxA10 and HoxA11 genes that control endometrial stromal cell decidualization. Moreover, HOXA10 depletion promotes cell invasion via increased expression of IL-6 and MMP and decreased expression of TIMP. There appears to be a negative correlation between IL-6 expression and decidualization. IL-6 was upregulated in endometrial stromal cells upon co-culture with activated macrophages. The cytokine IL-6 and its downstream ERK/MAPK signalling constitute a key pathway in adenomyosis through activation of proliferation and invasion of endometrial stromal cells and suppression of decidualization process.	[82,98-103]
NFKB1	nuclear factor kappa B subunit 1	NFKB1 is a transcription regulator that is activated by various intra- and extra-cellular stimuli and acts as a proinflammatory and immunomodulatory signaling molecule. NF-κB stimulates the expression of genes widely involved in cell survival, proliferation, apoptosis, angiogenesis, and fibrosis as a downstream transcription factor of IL1R and TNFR. Inappropriate activation of NFKB1 contributes to a number of inflammatory diseases and is characterized by the production of inflammatory cytokines. NF-κB p65 subunit is upregulated in ectopic and eutopic endometrial stromal cells in adenomyosis compared with	[3,64,104-106]

		normal endometrium. Suppression of NFKB1 gene expression inhibits embryo implantation through downregulation of LIF expression. Immunohistochemistry revealed that nuclear p65 expression was negatively associated with PR-B expression, and was associated with the severity of dysmenorrhea and abnormal uterine bleeding associated with adenomyosis.	
IL22	interleukin 22	IL-22, a member of the IL-10 family, plays an essential role in maintaining skin and mucosal homeostasis. IL-22 promotes the invasiveness of human endometrial stromal cells and angiogenesis of vascular endothelial cells through stimulating the production of IL-6, IL-8, RANTES, and VEGF. IL-22 secreted by decidual stromal cells and NK cells promotes the survival of human trophoblasts via inhibiting apoptosis and participates in successful establishment and maintenance of pregnancy. IL-22 and its receptors in the ectopic and eutopic endometrium of adenomyosis were significantly higher than those of normal endometrium. IL-22 has been reported to promote endothelial cell angiogenesis and stromal cell invasion. Aberrant expression of IL-22 and its receptors may play crucial roles in the pathogenesis of adenomyosis. Therefore, targeted inhibition of IL-22 may represent a potential treatment strategy for adenomyosis.	[107-109]
PTGS2	prostaglandin-endoperoxide synthase 2, also known as COX-2	Prostaglandin-endoperoxide synthase (PTGS), also known as cyclooxygenase (COXs), is involved in prostaglandin biosynthesis. COX-2 and lipoxygenase-5 (LOX-5) are upregulated in the ectopic and eutopic endometrial stromal cells in adenomyosis compared with normal endometrial stromal cells. The expression of COX-2 and LOX-5 showed positive correlations with that of IL-6 and IL-8, suggesting that inflammatory pathological conditions play an important role in adenomyosis predisposition. Indeed, the COX-2 expression was positively correlated with the severity of dysmenorrhea. The decidualization process is suppressed by inhibition of Cox-2 expression. In addition, COX-2, a	[110,111]

		downstream target of NF-kB, promotes decidualization of endometrial stromal cells.	
CCL5	C-C motif chemokine ligand 5, also known as RANTES (regulated upon activation, normal T-cell expressed and secreted)	The CCL5 chemokine, a member of the C-C motif chemokine subfamily, functions as a chemoattractant for blood monocytes and memory T helper cells. CCL5 is involved in immunoregulatory and inflammatory processes such as the accumulation of immune cells. RANTES affects the decidualization of endometrial stromal cells and enhances trophoblast invasion during early human placentation. Moreover, the RANTES expression was correlated with the severity of dysmenorrhea in women with deep infiltrating endometriosis. In addition, ectopic endometrium from women with adenomyosis has increased expression of RANTES compared with normal endometrium from healthy women.	[90,112]
